# Production of Human Cu,Zn SOD with Higher Activity and Lower Toxicity in* E. coli* via Mutation of Free Cysteine Residues

**DOI:** 10.1155/2017/4817376

**Published:** 2017-02-16

**Authors:** Kun Zhang, Yuejuan Zhang, Jing Zi, Xiaochang Xue, Yi Wan

**Affiliations:** ^1^Microbiology Institute of Shaanxi, Xi'an 710043, China; ^2^State Key Laboratory of Cancer Biology, Department of Biopharmaceutics, School of Pharmacy, Fourth Military Medical University, Xi'an 710032, China

## Abstract

Although, as an antioxidant enzyme, human Cu,Zn superoxide dismutase 1 (hSOD1) can mitigate damage to cell components caused by free radicals generated by aerobic metabolism, large-scale manufacturing and clinical use of hSOD1 are still limited by the challenge of rapid and inexpensive production of high-quality eukaryotic hSOD1 in recombinant forms. We have demonstrated previously that it is a promising strategy to increase the expression levels of soluble hSOD1 so as to increase hSOD1 yields in* E. coli*. In this study, a wild-type hSOD1 (wtSOD1) and three mutant SOD1s (mhSOD1s), in which free cysteines were substituted with serine, were constructed and their expression in soluble form was measured. Results show that the substitution of Cys111 (mhSOD1/C111S) increased the expression of soluble hSOD1 in* E. coli* whereas substitution of the internal Cys6 (mhSOD1/C6S) decreased it. Besides, raised levels of soluble expression led to an increase in hSOD1 yields. In addition, mhSOD1/C111S expressed at a higher soluble level showed lower toxicity and stronger whitening and antiradiation activities than those of wtSOD1. Taken together, our data demonstrate that C111S mutation in hSOD1 is an effective strategy to develop new SOD1-associated reagents and that mhSOD1/C111S is a satisfactory candidate for large-scale production.

## 1. Introduction

Human Cu,Zn superoxide dismutase 1 (hSOD1) is an antioxidant enzyme which protects cells against the oxidative by-products of aerobic metabolism through catalyzing the dismutation of superoxides into oxygen and hydrogen peroxide [1–3]. Given the potential importance of hSOD1 in medical applications such as limiting the detrimental effects of reactive oxygen species (ROS) in ischemia-reperfusion injury [4] and protecting cells from radiotherapy [5, 6], the development of efficient and viable production systems to obtain hSOD1 with higher yields or activity has received much attention for many years [7, 8].

Although the* E. coli* expression system is one of the most widely used systems for large-scale production of recombinant proteins, expressed proteins often form inclusion bodies without biological activity, which thus puts a constraint to limit their yields [9–11]. To remove this constraint, we have previously expressed hSOD1 in* E. coli* and purified it from both the inclusions and the cell lysate supernatant. The specific activity of the recombinant hSOD1 purified from the supernatant was higher than that from the inclusions by approximately 3-fold (unpublished data). In view of this, we assume that increasing the hSOD1 soluble expression level in* E. coli* may probably improve its specific activity and yields.

Disulfide bond exchange and intermolecular disulfide bond formation can both produce insoluble protein aggregates and affect protein activity [12]. For example, the EC1 domains of E-cadherins with a single free Cys residue can form a covalent dimer, thus inducing the precipitation of the proteins [13]. hSOD1 is a dimeric metalloprotein composed of identical non-covalently linked subunits [14], each of which contains 4 cysteine residues. Cys57 and Cys148 form an intrasubunit disulfide bond whereas Cys6 and Cys111 remain unbonded. Cys6 is buried within the protein whereas Cys111 is on its surface near the dimer interface [15, 16]. It has been shown that incorrect localization of cysteine in hSOD1 has caused protein oligomerization in ALS mice [17]. Cozzolino et al. studied the mechanism of the aberrant aggregation of mutant SOD1 (mutSOD1), which is closely associated with familial amyotrophic lateral sclerosis (FALS) [18]. Through mutating each of the four cysteine residues in SOD1 and analyzing the solubility and aggregation of those SOD1s in NSC-34 cells, they found that covalent disulfide cross-linking plays a pivotal role in the production of mutSOD1 aggregates and removal of Cys111 remarkably reduces the ability of FALS-related mutSOD1s to form aggregates and improves the viability of NSC-34 cells. However, considering that the recombinant protein expression efficiency and redox environment of microorganisms differ from those of mammalian cells, it is still unknown whether the cysteine mutation in hSOD1 can reduce aggregates formation and improve soluble expression in* E. coli*.

Given these observations, in the present study, we constructed three hSOD1 mutants, in which free cysteines were substituted with serine. Two single cysteine mutants were produced by replacing either Cys6 (mhSOD1/C6S) or Cys111 (mhSOD1/C111S) with serine, and one double cysteine mutant was produced by replacing both Cys6 and Cys111 with serine (mhSOD1/C6S/C111S). The three mutants and the wild-type hSOD1 (wtSOD1) were expressed in* E. coli*, and the soluble protein fractions and protein yields of the enzyme were evaluated. As misfolded or aggregated hSOD1 is toxic to mammalian cells [19], the toxicity and activity of mhSOD1/C111S with the highest level of soluble expression and yields were also evaluated. All the experiments were performed to determine the mutant hSOD1 with higher soluble expression but lower toxicity and stronger activities and to find the optimal candidate for large-scale production of hSOD1.

## 2. Materials and Methods

### 2.1. Materials

Restriction endonucleases, Taq polymerase, T4 DNA ligase, Pfu DNA polymerase, and MiniBEST Universal Genomic DNA Extraction Kit were purchased from TaKaRa (Dalian, China). Urea, *β*-mercaptoethanol, and Tris-HCl were purchased from Serva (Heidelberg, Germany). DEAE-Sepharose Fast Flow system was purchased from GE Healthcare (Wikströms, Sweden). Mouse anti-hSOD1 monoclonal antibody was purchased from Santa Cruz Biotechnology (California, USA). HRP-conjugated goat anti-mouse IgG was purchased from Zhongshan Golden Bridge Biotechnology Co. Ltd. (Beijing, China). The pET-22b (+) expression vector was purchased from Novagen (Madison, WI). The native hSOD1 gene was synthesized by Shenggong Company (Shanghai, China). All the chemicals were of analytical grade. RPMI 1640 medium and FBS were purchased from Life Technologies (Carlsbad, CA). Trypsin and MTT were purchased from Sigma (St. Louis, MO).

### 2.2. Construction and Expression of Recombinant hSOD1 and Its Mutants

The genes of hSOD1 (accession number 134611) and mutant SOD1s including mhSOD1/C6S, mhSOD1/C111S, and mhSOD1/C6S/C111S were synthesized by Shenggong Company.

With DNA sequencing confirmed, all the genes were digested with* NdeI* and* SalI *and cloned into pET22b (+) prokaryotic expression plasmid. Then the constructed plasmids were transformed into* E*.* coli *BL21 (DE3) competent cells. To induce SOD1 expression, positive BL21 (DE3) clones were grown overnight in 10 mL of LB medium supplemented with ampicillin (100 *μ*g/mL) at 200 rpm shaking and 37°C. Afterwards, 2 mL of each culture was transferred to 200 mL of fresh LB in 500 mL flasks. The cultures were incubated at 37°C and 200 rpm until the OD_600_ reached 0.5 when they were induced with 1 mM IPTG. The cells were harvested 4 h later by centrifugation at 12,000*g* for 10 min at 4°C and stored at −20°C. Finally, 1 mL of the culture was collected and analyzed by SDS-PAGE.

### 2.3. Analysis of the Levels of Soluble Expression of hSOD1 and mhSOD1s

Five grams of harvested cells was washed in PBS twice, resuspended in 50 mL of lysis buffer (10 mM Tris and 1 mM EDTA, pH 8.0), and sonicated on ice (20 min in all, intervals of 5 s on/10 s off, power output of 300 W). Then, 100 *μ*L of the lysates was centrifuged at 12,000*g* for 30 min at 4°C. The supernatant was collected as the soluble fraction, whereas the pellets (collected as insoluble fraction) were washed in PBS and resuspended in 100 *μ*L of Laemmli sample buffer (62 mM Tris-HCl, pH 6.8, 10% glycerol, 2% SDS, 5%  *β*-mercaptoethanol, and 0.05% bromophenol blue). After the samples were subjected to 15% SDS-PAGE and transferred to nitrocellulose membranes (0.22 *μ*m, Invitrogen, USA), Western blot analysis was carried out. An ECL chemiluminescence system (Pierce, Rockford, USA) was used for detection. Results of the 4 independent experiments were quantified with BandScan 5.0 image analysis software.

### 2.4. Purification of hSOD1s

Ten grams of induced wet* E. coli* cells expressing wtSOD1, mhSOD1/C6S, mhSOD1/C111S and mhSOD1/C6S/C111S was suspended in equal volumes of lysis buffer and disrupted by sonication. The supernatant was collected by centrifugation at 12,000 rpm for 20 min at 4°C. Then, with 1 mM CuSO_4_ added to the supernatant, the samples were heated at 70°C for 15 min. After the precipitated proteins were removed by centrifugation, the supernatant was thoroughly dialyzed against a volume 30 times that of buffer *A* (10 mM Tris-HCl pH 8.0) for 24 h and the insoluble particles were removed by filtering through a 0.22 *μ*m syringe filter (VWR, West Chester, PA). A DEAE-Sepharose 26 × 200 mm column with 20 mL of column volume (CV) was equilibrated by buffer *A* with 5 CV. The samples were then loaded onto the column at a speed of 1 mL/min (ÄKTA purifier, GE Healthcare) at room temperature. The column was eluted with a linear gradient of NaCl from 0 to 1 M at a flow rate of 1 mL/min with 10 CV of buffer *B* (buffer *A* + 1.0 M NaCl, pH 8.0). After the purified proteins were dialyzed against ddH_2_O for three times, they were frozen and lyophilized. Purity of the proteins was monitored by SDS-PAGE and HPLC.

### 2.5. Characterization of hSOD1s

The activity of the purified SOD1 proteins was determined as previously described [20]. In brief, 150 *μ*L of 4.5 mM pyrogallol was added to 4.35 mL Tris-HCl buffer (0.1 M Tris, 1 mM EDTA·2Na, and pH 8.2) for autoxidation, and the absorbance was assessed at a wavelength of 325 nm (OD_325_) with the autoxidation rate modulated to approximately 0.06 OD min^−1^. SOD1 activity was measured by monitoring the change in OD_325_ resulting from the inclusion of 20 *μ*L of the sample in the 4.35 mL Tris-HCl buffer solution prior to the addition of pyrogallol. Generally, a unit of the enzyme is defined as the amount of the enzyme which inhibits the pyrogallol autoxidation rate by 50% per min.

The amounts of the metals copper and zinc were measured using a novAA300 atomic absorption spectrometer (Analytik Jena AG, Germany) [21].

### 2.6. Analysis of hSOD1s Toxicity

Converging evidence indicates that misfolded or aggregated hSOD1 is toxic to mammalian cells through several ways [19, 22–24]. To evaluate the toxicity of the wtSOD1 and mhSOD1/C111S, Baby Hamster Kidney (BHK) cells suspended in RPMI 1640 medium (8 × 10^3^ cells in 200 *μ*L) were seeded in 96-well culture plates in triplicate and treated with serially diluted SOD1s for 1–5 days. Bovine serum albumin (BSA) was used as the negative control. The cell numbers were measured by Cell Counting Kit-8 (BioTek Inc., VA) according to the manufacturer's instruction. The cell proliferation rate was calculated from the cell numbers and normalized to BSA-treated cells.

To further investigate the toxicity of endogenous mhSOD1/C111S, both wtSOD1 and mhSOD1/C111S genes were cloned into pcDNA3 (Invitrogen) plasmid and transfected into BHK cells with Lipofectamine 2000 according to standard instructions. Cell growth rate was calculated at 24 and 48 h after transfection. To evaluate the solubility and aggregation of SOD1s, BHK cells were collected and the supernatant (soluble fraction) and the pellet (insoluble fraction) samples were prepared according to a previously described method [18]. Finally, soluble and insoluble SOD1s expressed in BHK cells were detected by Western blot.

### 2.7. Whitening Effects of hSOD1s

The whitening effects of wild-type and mutant SOD1s were measured by their modulation of melanocytic proliferation and tyrosinase activity according to standard methods.

To detect the effects of SOD1s on melanocytic proliferation, 3-4 × 10^3^ mouse skin melanoma B16 cells were seeded in 96-well plates in triplicate and cultured for 48 h at 37°C under 5% CO_2_ in humid air. Next, SOD1s were added and cell proliferation was analyzed by MTT assay as previously described [25]. In brief, the cells were incubated for 1, 2, or 3 days, respectively, and 20 *μ*L of MTT (0.5 mg/mL) was added. Then, the plates were centrifuged (2000 rpm for 10 min) 4 h later and the supernatants were discarded. Finally, 100 *μ*L of DMSO was added and the solubilized MTT was measured at 570 nm using a Bio-Rad plate reader and the cell growth rate was calculated with the formula: Growth rate (%) = OD sample/OD control × 100%.

The in vitro tyrosinase inhibition assays were performed according to the method described previously [26, 27]. In brief, 30 *μ*L of mushroom tyrosinase (final concentration 6.67 *μ*g/mL) was preincubated with serially diluted wtSOD1 and mhSOD1/C111S (1.25, 2.5, 3.75, 5.0, 7.5, and 10.0 mg/mL) in 50 mM NaHPO_4_-NaH_2_PO_4_ buffer (pH 6.8) for 10 min at 30°C. The substrate L-DOPA (dihydroxyphenylalanine, 0.5 mM) was added to the mixture and formation of the L-dopaquinone was continuously monitored by assaying the change in absorbance at 475 nm for 1 min. The activity was expressed as the sample concentration, which inhibited 50% of the enzyme activity (IC50). The percentage of inhibition of tyrosinase was calculated as follows: Inhibition rate (%) = (*A* − *B*)/*A* × 100%, where *A* and *B* represent the absorbance values for the blank and for the samples, respectively.

The in vivo tyrosinase inhibition was assayed by using B16 cells. In brief, 3-4 × 10^3^ cells were seeded in 96-well plates and cultured for 24 h at 37°C. Then, wtSOD1 and mhSOD1/C111S serially diluted with RPMI 1640 (0.0039, 0.0156, 0.0625, 0.25, and 1 mg/mL) were added and the cells were cultured for another 48 h. RPMI 1640 medium was used as the negative control. After being washed with cold PBS twice, cells were lysed in 90 *μ*L 1% Triton X-100 (in PBS) by freezing. Finally, 10 *μ*L L-Dopa (0.1%) was added and incubated at 37°C for 1 h and the absorbance at 475 nm was assayed to calculate the inhibition percentage of tyrosinase as follows: Inhibition rate (%) = (1−(OD sample ÷ OD control)) × 100%.

### 2.8. Determination of the Antiradiation Activity of hSOD1s

One of the most important functions of hSOD1 is that it can resist radiation. To test the antiradiation activities of SOD1s, wtSOD1 and mhSOD1/C111S were used to treat BHK cells under ultraviolet (UV) radiation and the protective effects of SOD1s on cell viability and DNA damage were measured.

To evaluate the protective effects of wtSOD1 and mhSOD1/C111S on UV irradiation, about 2 × 10^5^ BHK cells in 200 *μ*L medium were seeded in 48-well plates and cultured for 24 h. Then, the cells were washed with PBS and incubated in 100 *μ*L of fresh RPMI 1640 medium containing 1% FBS. Afterwards, wtSOD1 and mhSOD1/C111S diluted with RPMI 1640 medium (0.0039, 0.0078, 0.0156, 0.0312, and 0.0625 mg/mL) were added after PBS washing. Kojic acid and arbutin were used as positive controls. Cells that received radiation or none in the absence of both wtSOD1 and mhSOD1/C111S were used as normalization controls. The protection ratio was calculated with the formula: (OD sample − OD radiative control)/(OD nonradiative control − OD radiative control) × 100%.

For DNA damage analysis, BHK cells were seeded in 6-well plates and treated as described above. After being irradiated with UV, the cells were cultured in a fresh medium for 12 h and washed with PBS once. Then, the cells were incubated with fresh PBS for 10 min and harvested by centrifugation. Finally, genomic DNA was extracted and the DNA damage was analyzed by agarose gel electrophoresis.

All the experiments were repeated 4 times and the medium without SOD1 was used as control groups.

### 2.9. Statistical Analysis

The statistical significance of the differences was evaluated using Student's two-tailed *t*-test. Differences were considered significant when *P* < 0.05.

## 3. Results and Discussion

In the previous work, for large-scale production of recombinant hSOD1, wtSOD1 was expressed in* E. coli*, in which soluble wtSOD1 accounted for approximately 36% of the total expressed protein. Then, wtSOD1 was purified from both the intracellular inclusions and the cell lysate supernatant. The specific activity of the protein purified from the cell lysate supernatant was approximately 6000 IU/mg, which was 3 times higher than that of the enzyme purified from the inclusion bodies (unpublished data). In view of these results, we expected that increasing the soluble expression levels of SOD1 in* E. coli* might effectively improve the yield of hSOD1. In this study, a method for substituting the free cysteines in SDO1 was used to increase the soluble expression and the yield of hSOD1 in* E. coli.*

### 3.1. Construction and Expression of Recombinant wtSOD1 and Its Mutants

**Figure 1:**
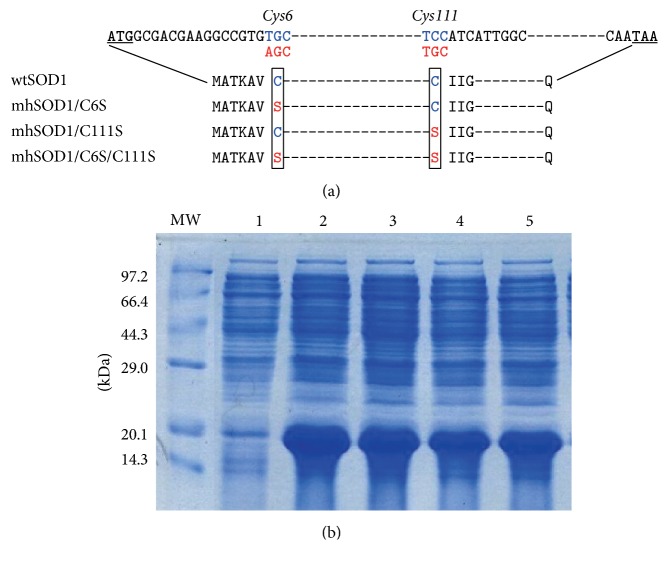
Construction and expression levels of wtSOD1 and its mutants in* E. coli*. (a) Schematic diagram of the molecular structure of the wild-type and mutant SODs constructed. Blue color indicates the wild-type DNA or amino acid sequences and red color indicates the mutant ones. (b) Expression of wtSOD1 and its mutants by SDS-PAGE analysis. Lane MW: molecular weight standard ladder (kDa). Lane 1: whole cell lysate of wtSOD1-expressing cells before induction. Lanes 2–5: whole cell lysate of cells expressing wtSOD1 (lane 2), mhSOD1/C6S (lane 3), mhSOD1/C111S (lane 4), and mhSOD1/C6S/C111S (lane 5) after induction.

All the DNA fragments encoding wtSOD1 and three mutants, in which Cys6 (mhSOD1/C6S), Cys111 (mhSOD1/C111S), or both Cys6 and Cys111 (mhSOD1/C6S/C111S) were substituted with serine, were synthesized by Shenggong Company (Figure 1(a)). The successful insertion of DNA fragments encoding wtSOD1 or its mutants into pET-22b(+) bacterial expression plasmids was confirmed by digestion with* NdeI* and* SalI* and DNA sequencing (data not shown). Then, wtSOD1 and its mutants were successfully induced in* E. coli* BL21 (DE3) when the transformed bacteria were treated by 1 mM IPTG (Figure 1(b)). Molecular weight of the expressed proteins was approximately 20.1 kDa, which is consistent with the results obtained by Hartman et al. [28] (Figure 1(b)). The recombinant proteins represented approximately 55% of the total bacterial protein, as is determined by densitometric scanning.

### 3.2. Comparison of Soluble Expression Levels of Recombinant SOD1s

**Figure 2:**
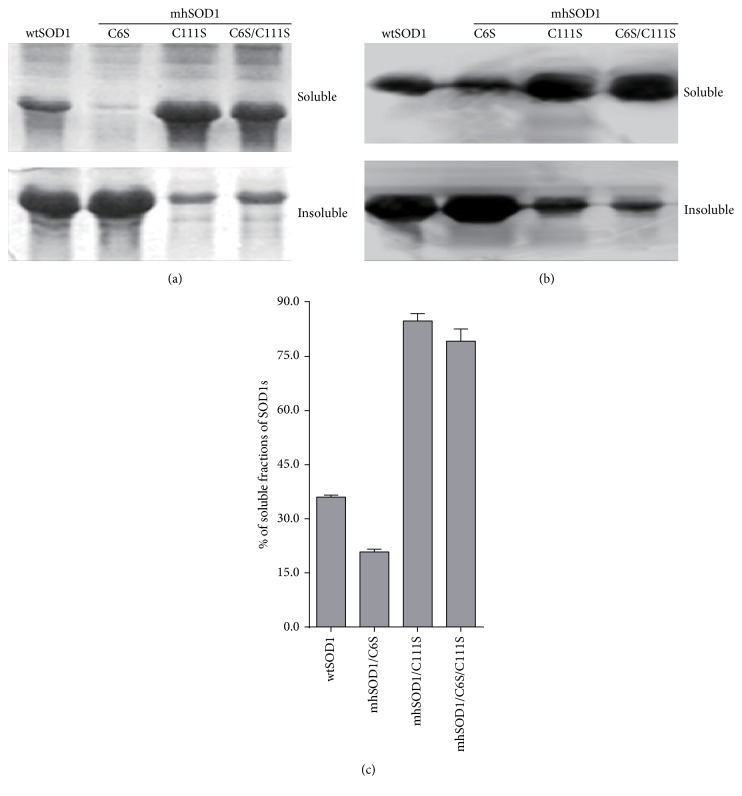
Effects of cysteine mutations on the solubility of hSOD1 expressed in* E. coli*. (a) SDS-PAGE of soluble and insoluble protein fractions. (b) Western blotting of soluble proteins. Proteins on the SDS-PAGE gel were transferred to membranes, blotted with an anti-SOD1 antibody to determine the relative percentage of the soluble fractions. The lanes contain wtSOD1 or three mutants as indicated. (c) Histogram of the distribution of the soluble fractions of SOD1 in* E. coli* as determined by 4 independent Western blot experiments.

Western blot results have confirmed that a hSOD1 monoclonal antibody could effectively detect the expressed wtSOD1 and all of its mutants (Figures 2(a) and 2(b)). The protein expressions in the soluble and insoluble fractions were compared. Soluble wtSOD1 accounted for approximately 36.1% of the total expressed SOD1 (Figure 2(c)). The single substitution of Cys6 (mhSOD1/C6S) decreased soluble SOD1 expression remarkably from 36.1% to 20.8% whereas the single substitution of Cys111 (mhSOD1/C111S) increased soluble expression significantly from 36% to 84.9%. No significant difference was found between the soluble protein expression levels of the double mutant mhSOD1/C6SC111S and that of mhSOD1/C111S.

These results suggest that Cys111 is a mediator essential to soluble expression. The observed decrease in the expression of soluble protein when Cys6 was substituted suggests that during the protein folding process, the inner free cysteine may prevent the formation of an unfavorable disulfide bond between Cys111 and either Cys57 or Cys148. This is supported by the expression level of the soluble protein, mhSOD1/C6S/C111S, which is similar to that of mhSOD1/C111S. The expression levels of the two soluble proteins are significantly higher than that of mhSOD1/C6S (Figure 2).

### 3.3. Comparison of the Protein Yield and Activity of Recombinant wtSOD1 with Its Mutants

**Figure 3:**
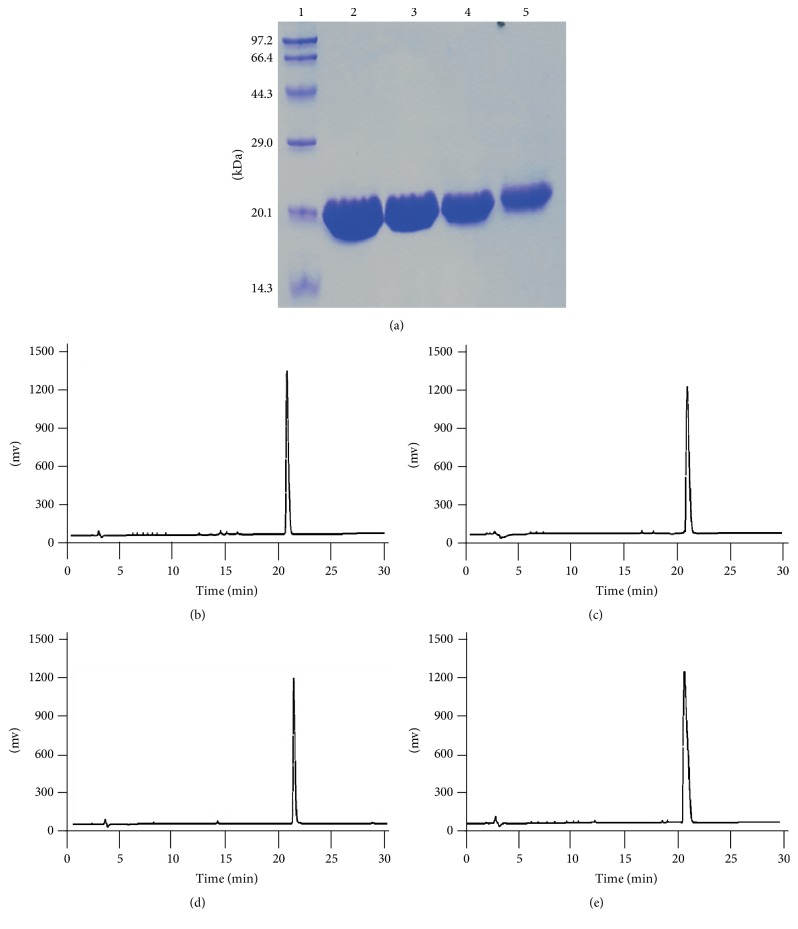
Purity analysis of SOD1s by SDS-PAGE and HPLC. (a) Purity of SOD1s was analyzed by SDS-PAGE. Lane 1: molecular weight standard ladder (kDa). Lanes 2–5: purified wtSOD1 (lane 2), mhSOD1/C6S (lane 3), mhSOD1/C111S (lane 4), and mhSOD1/C6S/C111S (lane 5). (b) HPLC analysis of purified wtSOD1 (a), mhSOD1/C6S (b), mhSOD1/C111S (c), and mhSOD1/C6S/C111S (d). A reverse-phase column was used for HPLC (Beckman, 250 × 5.0 mm ID, Kromasil C18, Akzo Nobel Co. Ltd. (Sweden)). 5 *μ*g samples in PBS were injected into the column and then eluted by linear gradient: 100% solution A (water, 5% acetone, and 0.1% TFA) to 40% solution B (acetone, 0.1% TFA) over 40 minutes with a flow rate of 1.0 mL/min. The eluted fractions were analyzed at 280 nm, and the purity of the proteins was calculated as a percentage of the total peak area.

The lytic supernatants of the cell cultures expressing recombinant wtSOD1, mhSOD1/C6S, mhSOD1/C111S, and mhSOD1/C6S/C111S were purified with DEAE-Sepharose Fast Flow columns. The purity of the isolated proteins was above 95% in all the cases, as confirmed by SDS-PAGE (Figure 3(a)) and HPLC (Figures 3(b)–3(e)). The activity yields of wtSOD1, mhSOD1/C6S, mhSOD1/C111S, and mhSOD1/C6S/C111S purified per gram of wet* E. coli* cells showed the following progression: mhSOD1/C111S and mhSOD1/C6S/C111S > wtSOD1 > mhSOD1/C6S (wtSOD1, 6.2 × 10^4^  ± 2.1 × 10^3^ IU; mhSOD1/C6S, 1.6 × 10^4^  ± 1.5 × 10^3^ IU; mhSOD1/C111S, 2.02 × 10^5^  ± 3.5 × 10^3^ IU; mhSOD1/C6S/C111S, 1.98 × 10^5^  ± 5.3 × 10^3^ IU). All these results collectively verified our hypothesis that an increase in the soluble expression levels may increase hSOD1 yields (Table 1). For this reason, mhSOD1/C111S was selected for our subsequent experiments to investigate the toxicity and activity of mhSOD1s, whereas wtSOD1 was used as the control.

Considering the presence of copper and zinc ions in the active site of SOD1 enzymes is critical for their activity, both copper and zinc contents of the purified proteins were determined. The results from the atomic absorption measurements of the purified proteins were showed in Table 2 and it showed that mhSOD1/C111S increased the metallization considerably.

### 3.4. Toxicity of Recombinant SOD1s

**Figure 4:**
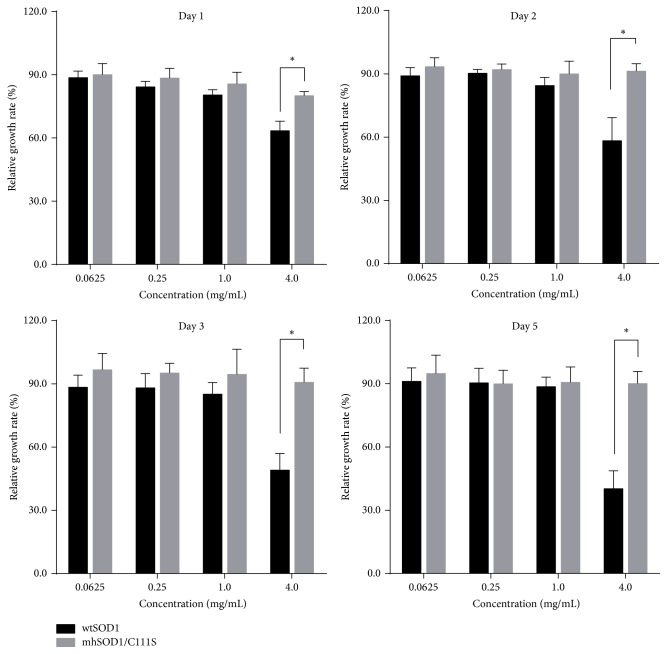
Toxicity of wtSOD1 and mhSOD1/C111S to BHK cells. BHK cells were treated with recombinant SOD1s, which were serially diluted with RPMI 1640 medium, for 1–5 days and cell proliferation was assessed by CCK-8 method. BSA was used as the negative control. ^*∗*^*P* < 0.05 compared between indicated groups. Data are representative of three experiments.

To determine whether the cysteine substitution can affect the toxicity of recombinant SOD1, wtSOD1 and mhSOD1/C111S were used to treat BHK cells and cell proliferation was assessed by CCK-8 solution at indicated time points. Bovine serum albumin (BSA) was used as the negative control in which the growth rate of the cells was set as 100%. As shown in Figure 4, neither wtSOD1 nor mhSOD1/C111S had hardly any impact on cell proliferation when the dosage was low (0.0625–1.0 mg/mL). As to higher dosage, 4.0 mg/mL wtSOD1 is apparently toxic to BHK cells and the relative growth rate of the cells is reduced by 50–60%, whereas the toxicity of mhSOD1/C111S is significantly lower than that of wtSOD1 (*P* < 0.05) since the cells treated with mhSOD1/C111S for 1–5 days maintained their normal proliferation. These data indicate that to BHK cells mhSOD1/C111S is safer than wtSOD1.

**Figure 5:**
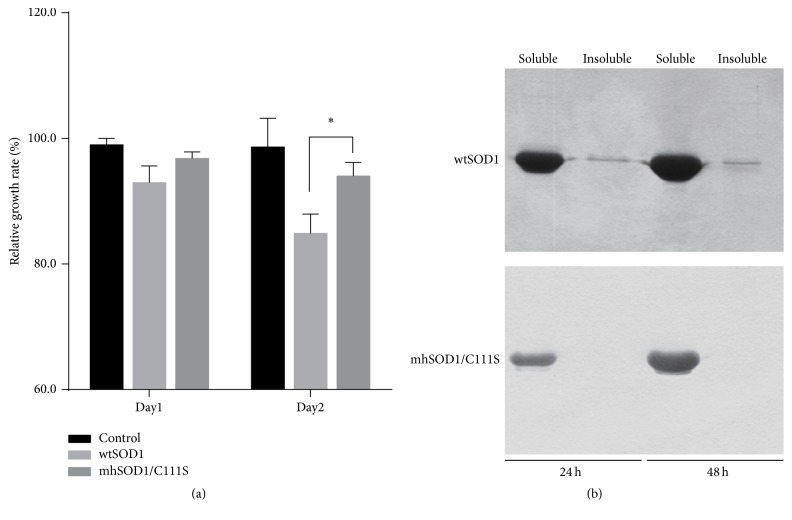
Toxicity of wtSOD1 and mhSOD1/C111S to BHK cells in vivo. BHK cells were transfected with recombinant plasmids pcDNA3-wtSOD1 and pcDNA3-mhSOD1/C111S by using Lipofectamine 2000. The empty plasmid pcDNA3 was used as the negative control. Cell proliferation was assessed by CCK-8 method and SOD1s expression as soluble and insoluble forms was detected by Western blot 24 h and 48 h after transfection. ^*∗*^*P* < 0.05 compared between indicated groups. Data are representative of three experiments.

To study the possible toxicity of mhSOD1/C111S in mammalian cells, the ability of mhSOD1/C111S to affect cell proliferation and SOD1 solubility was tested in a cell transfection assay. As shown in Figure 5(a), wtSOD1 transfection remarkably inhibited whereas mhSOD1/C111S has no apparent effect on BHK cell proliferation. As to SOD1 solubility, only a subtle aggregation was detected in wtSOD1-transfected BHK cells, but almost all mhSOD1/C111S is accumulated in the soluble form and no aggregation was found (Figure 5(b)). Both wtSOD1 and mhSOD1/C111S are more soluble in mammalian cells than* E. coli*, and it is most probably because of a more powerful chaperone system and more effective redox environment in mammalian cells.

### 3.5. Whitening Effects of SOD1

**Figure 6:**
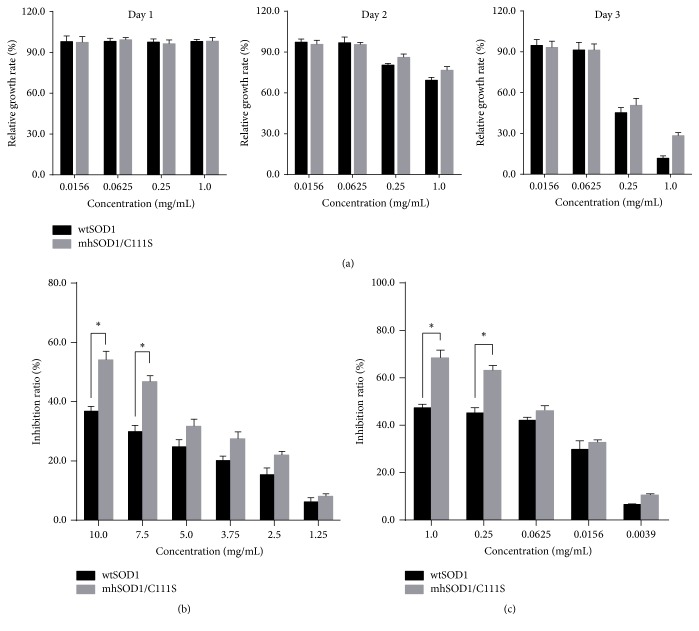
Whitening effects of wtSOD1 and mhSOD1/C111S. (a) B16 cells were treated with both wtSOD1 and mhSOD1/C111S diluted with RPMI 1640 medium and cell proliferation was analyzed by MTT assay. ((b) and (c)) The inhibition of mushroom tyrosinase activity by wtSOD1 and mhSOD1/C111S was measured in vitro (b) and in vivo (c). ^*∗*^*P* < 0.05 compared between indicated groups.

To investigate the whitening effects of SOD1s, we first assessed the effects of SOD1s on B16 melanocyte proliferation. Results showed that both wtSOD1 and mhSOD1/C111S apparently inhibit B16 cell proliferation at higher dosages (0.25–1 mg/mL) on days 2 and 3 (Figure 6(a)). Next, we detected the inhibitory effects of SOD1s on tyrosinase. As shown in Figures 6(b) and 6(c), both wtSOD1 and mhSOD1/C111S apparently inhibited tyrosinase activity in vitro and in vivo. Interestingly, the effects of mhSOD1/C111S are stronger than that of wtSOD1 in the cell-free in vitro system and the in vivo system. We conjecture that this is probably due to the fact that the conformation or half-life of mhSOD1/C111S is longer than that of wtSOD1. The detailed mechanism of this is still under investigation. In addition, we found that the effects of both wtSOD1 and mhSOD1/C111S on melanocytic proliferation and mushroom tyrosinase are not only time-dependent but also dose-dependent.

### 3.6. Antiradiation Activity of SOD1

**Figure 7:**
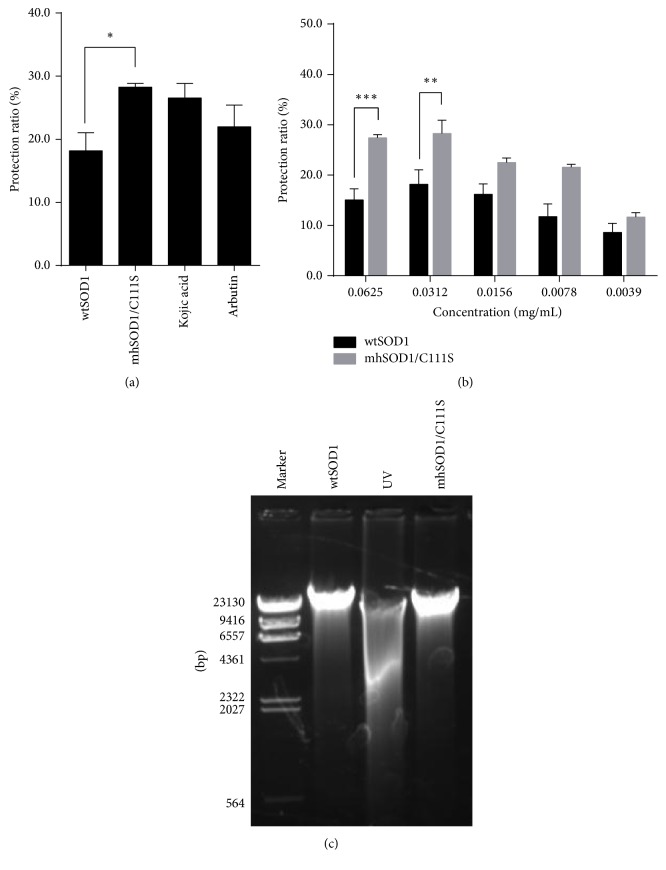
Antiradiation effects of SOD1s. (a) BHK cells were treated with wtSOD1 and mhSOD1/C111S and cell proliferation was analyzed by MTT assay. Kojic acid and arbutin were used as positive controls. (b) BHK cells were treated with serially diluted wtSOD1 and mhSOD1/C111S and cell proliferation was analyzed by MTT assay. ^*∗*^*P* < 0.05; ^*∗∗*^*P* < 0.01; ^*∗∗∗*^*P* < 0.001 compared between indicated groups. (c) BHK cells were treated with wtSOD1 and mhSOD1/C111S for 3 h, followed by UV radiation for 20 min, and DNA damage was analyzed by agarose gel electrophoresis. Data are representative of three experiments.

To test the antiradiation activity of SOD1s, wtSOD1 and mhSOD1/C111S were used to treat BHK cells under UV radiation and the protective effects of SOD1s on cell viability and DNA damage were measured.  Figure 7(a) shows that mhSOD1/C111S has a stronger antiradiation effect than wtSOD1 (*P* < 0.05). The protective effect of mhSOD1/C111S on UV-treated BHK cells is even slightly better than that of kojic acid and arbutin. When higher dosages (0.0625 and 0.0312 mg/mL) are given, significant differences were found between mhSOD1/C111S- and wtSOD1-treated cells (*P* < 0.001 and *P* < 0.01, resp.) with the former having a stronger activity (Figure 7(b)). We further detected DNA damage in UV-radiated BHK cells. As shown in Figure 7(c), both wtSOD1 and mhSOD1/C111S treatments effectively prevented DNA damage in BHK cells when compared with UV-radiated cells.

## 4. Conclusions

As a soluble cytoplasmic and mitochondrial intermembrane superoxide dismutase, hSOD1 can prevent damage to cell components caused by free radicals derived from aerobic metabolism. Although some SOD1 drugs have been developed for clinics to treat a wide spectrum of human diseases, including Peyronie's disease [29], rheumatoid arthritis [30], and acute lung injury [31], without demonstrable toxicities [32], unfortunately, large-scale manufacturing and clinical use of hSOD1 are still limited because of the challenge of rapid and inexpensive production of high-quality hSOD1 in recombinant forms. Thus, great attention has been given to the development of efficient and viable production systems to obtain hSOD1 with higher yields or activity [7, 8]. Eukaryotic expression systems are suitable systems for producing hSOD1 and dozens of groups have made progress on SOD1 preparation [18, 33–35]. For example, Cozzolino et al. successfully expressed mutant hSOD1 in NSC-34 cells and confirmed that removal of Cys111 remarkably improves the solubility of SOD1 [18]. However, it is difficult to be industrialized because the expression efficiency is low and thus the cost of production is high and on top of that the mammalian cell handling process is complex.

Although* E. coli* usually expresses recombinant protein more efficiently, the congenital deficiency in redox environment of* E. coli* makes it often express proteins as inclusion bodies and it is time-consuming to refold the products. As a result, increasing the soluble expression levels of recombinant proteins is a promising strategy that can increase the yields in* E. coli*. To study the role of the free cysteines of recombinant hSOD1 in soluble expression and yield in* E. coli*, we constructed and expressed three mutant hSOD1s, in which free cysteines were substituted with serines. We have verified that it is an effective stabilization strategy to replace cysteine with serine in hSOD1 so as to prevent the formation of unwanted intermolecular or intramolecular disulfide bonds and to raise the soluble protein expression levels in* E. coli. *Among these mutant SOD1s, the substitution of Cys111 (mhSOD1/C111S) increased significantly soluble hSOD1 expression in* E. coli *(the soluble expression of mhSOD1/C111S is 2.35 times that of wtSOD1). The substitution of the internal Cys6 residue, however, reduced significantly the expression of soluble protein (soluble expression of mhSOD1/C6S is 0.58 times that of wtSOD1). Although the only substitution of Cys6 reduced soluble expression remarkably, an overall increase can be observed (soluble expression of mhSOD1/C6S/C111S is 2.2 times that of wtSOD1) when Cys111 is simultaneously substituted with another amino acid. The purified protein yields of wild-type and mutant hSOD1s were also measured. These results proved that increasing soluble expression levels can increase hSOD1 yields. In addition, the mhSOD1 with higher soluble expression levels also produced stronger activity than the wtSOD1. The recombinant mhSOD1/C111S showed lower toxicity but stronger whitening and antiradiation activities than those of wtSOD1. All these data collectively demonstrate that substitution of cysteine with serine in human SOD1 is an effective strategy to develop new SOD1-associated reagents and that mhSOD1/C111S is a promising candidate for large-scale production.

## Figures and Tables

**Table 1:** Ratios of mhSOD1s to wtSOD1 in soluble expression and yields.

	wtSOD1	mhSOD1/C6S	mhSOD1/C111S	mhSOD1/C6S/C111S
Soluble expression	1.00	0.58 ± 0.02↓	2.35 ± 0.07↑	2.20 ± 0.10↑
Activity yield	1.00	0.26 ± 0.02↓	3.25 ± 0.07↑	3.19 ± 0.04↑

The ratios of mhSOD1s to wtSOD1 in soluble expression and yields were calculated. ↑ presents increase and ↓ presents decrease when compared with the wtSOD1.

**Table 2:** Atomic absorption measurements of Cu and Zn contents in SOD1 proteins.

SOD protein	Cu content		Zn content	
	*μ*g/mg	Cu/subunit^a^	*μ*g/mg	Zn/subunit^a^
wtSOD1	4.346	1.37	2.787	0.85
mhSOD1/C111S	5.322	1.67	3.822	1.16

^a^The molecular weight of wtSOD1 and mhSOD1/C111S is calculated as 20 kDa.
